# Anti-inflammatory response following uptake of apoptotic bodies by meningothelial cells

**DOI:** 10.1186/1742-2094-11-35

**Published:** 2014-02-24

**Authors:** Jia Li, Lei Fang, Peter Meyer, Hanspeter E Killer, Josef Flammer, Albert Neutzner

**Affiliations:** 1 Department of Biomedicine, University Basel, Basel, Switzerland; 2 Department of Ophthalmology, The Second Hospital of Jilin University, Changchun, China; 3 Department of Ophthalmology, University Basel, Basel, Switzerland; 4 Department of Ophthalmology, Kantonsspital Aarau, Aarau, Switzerland

**Keywords:** Meningothelial cells, Apoptotic cells, Cytokines, Cerebrospinal fluid, Central nervous system, Optic nerve

## Abstract

**Background:**

Meningothelial cells (MECs) are the cellular components of the meninges. As such, they provide important barrier function for the central nervous system (CNS) building the interface between neuronal tissue and the cerebrospinal fluid (CSF), and are also part of the immune response of the CNS.

**Methods:**

Human, immortalized MECs were analyzed by flow cytometry and confocal microscopy to study the uptake of apoptotic cells. Furthermore, cytokine and chemokine production by MECs was analyzed by cytokine array and ELISA.

**Results:**

We found that MECs are highly active phagocytes able of ingesting and digesting large amounts of apoptotic cells. Furthermore, the uptake of apoptotic cells by MECs was immune suppressive via inhibiting the secretion of pro-inflammatory and chemoattractant cytokines and chemokines IL-6, IL-8, IL-16, MIF, and CXCL1, while increasing the secretion of anti-inflammatory IL-1 receptor antagonist by MECs.

**Conclusion:**

MECs respond with the secretion of anti-inflammatory cytokines and chemokines following the uptake of apoptotic cells potentially connecting these cells to processes important for the shut-down of immune responses in the brain.

## Introduction

The central nervous system (CNS) is enveloped by a complex layered structure, the meninges, providing protection from outside influence. The meninges comprise dura mater and the leptomeninges, which consist of the arachnoid mater and pia mater together with the trabeculae and septae that traverse the cerebrospinal fluid (CSF-)filled subarachnoidal space [1,2]. The cellular component of the pia, arachnoid, and the trabeculae and septae of the subarachnoidal space are meningothelial cells (MECs) also referred to as leptomeningeal cells [3]. MECs form a monolayer and are connected via tight junctions, gap junctions, and desmosomes contributing as part of the pia mater to the barrier between the CSF and the neuronal tissue [4].

Recent work by us and others revealed several functions of MECs beyond their function as cells simply covering neuronal tissue. MECs were shown to be actively involved in physiological and pathophysiological processes in the subarachnoidal space and in the CSF compartment [5-7]. Namely, MECs were shown to influence subarachnoidal architecture during glaucomatous neurodegeneration through increased proliferation [8] and to modulate CSF composition under pathological conditions through the production of lipocalin-type prostaglandin D synthase [9]. Furthermore, MECs release pro-inflammatory cytokines under pathophysiologically relevant conditions such as oxidiative stress, increased pressure, or exposure to lipopolysaccharide [5]. In addition, MECs proved to be highly active facultative phagocytes capable of ingesting large amounts of particulate matter such as latex beads [5], but also Gram-positive and Gram-negative bacteria [10]. Thus, MECs can be considered a part of the CNS immune system.

Programmed cell death or apoptosis is essential for maintaining tissue homeostasis during development [11] and also occurs as part of immune cell maturation and during the termination of the immune response [12]. As final step of the apoptotic program, the correct disposal of apoptotic bodies via phagocytosis is essential, as apoptotic cells might otherwise be a source for autoantigens [13]. Thus, engulfment of apoptotic bodies is a mechanism to prevent otherwise detrimental inflammatory responses leading to autoimmune disease. Interestingly, uptake of apoptotic cells by phagocytes was shown to have a direct anti-inflammatory effect by blunting pro-inflammatory cytokine secretion of phagocytic cells further preventing inflammation as response to apoptotic cells [14].

Here we found that human MECs are capable of ingesting apoptotic cells efficiently via the endosomal route for digestion in the lysosomal compartment. Furthermore, MECs react to the uptake of apoptotic cells by decreasing their pro-inflammatory cytokine and chemokine secrection, while at the same time increasing the production of anti-inflammtory molecules suggesting that MECs are able to act anti-inflammatory and are involved in keeping immune-reactions in the CNS in check.

## Materials and methods

### Cell culture and treatments

Human WHO grade I meningioma derived, hTERT immortalized (Ben-Men-I) cells [15] were cultured in DMEM supplemented with 10% FCS, 2 mM L-glutamine, and 1 mM sodium pyruvate (Sigma-Aldrich). Primary porcine meningothelial cells (PMECs) were cultured as described previously by us [16]. Monocyte-like U-937 cells (Sigma-Aldrich, 85011440) were cultured in RPMI 1640 supplemented with 10% FCS, 2 mM L-glutamine, and 1 mM sodium pyruvate (Sigma-Aldrich). SH-SY5Y cells were cultured in DMEM supplemented with 15% FCS, 2 mM L-glutamine, and 1 mM sodium pyruvate. Ben-Men-I cells were treated with 0, 50, or 100 ng/mL of IL-6 (Sigma-Aldrich, SRP3096) or IL-8 (Sigma-Aldrich, I1645). For uptake studies, cells were incubated for 4 or 14 h with Alexa Fluor 488-labeled, heat- or chemically-killed *Staphylococcus aureus* (Invitrogen, S-23371) or apoptotic cells.

### Generation of fluorescently-labeled apoptotic bodies

1 × 10^6^ live U-937 or SH-SY5Y cells were stained with 0.5 μM CellTrace CSFE (Invitrogen, C34554) or CellTrace Violet (Invitrogen, C34557) in PBS for 15 min at 37°C in the dark, incubated for 30 min in complete medium at 37°C, washed. and resuspended in PBS. More than 99% of the cells were stained with CFSE as evaluated by flow cytometry (Additional file 1: Figure S3B). 10^6^ CFSE-labeled U-937 cells were resuspended in 10 mL of complete medium and treated with 700 ng/mL actinomycin D (Sigma-Aldrich, A9415) for 20 h and washed twice with PBS. SH-SY5Y cells were treated with 900 ng/mL actinomycin D for 24 h to induce apoptosis. Using annexin V staining and propidium iodide exclusion, induction of apoptosis was confirmed (Additional file 1: Figures S3C).

### Flow cytometric analyses

For the determination of phagocytic activity, Ben-Men-I cells were seeded at 5 × 10^4^ cells and grown in 3 mL of media in six well flat-bottom cell culture plates for 24 h. Following incubation with CFSE-labeled apoptotic U-937 or SH-SY5Y cells at the indicated ratios, cells were harvested using trypsin/EDTA, washed three times with FACS buffer (10 mM EDTA, 1% FBS in PBS), resuspended in FACS buffer before acquisition using a CyAnADP flow cytometer (Beckman Coulter).

### Microscopy

For detection of phagocytosis, Ben-Men-I cells or PMECs were grown on glass cover slips for 24 h, incubated with CFSE-labeled apoptotic cells for 4 h, washed five times with PBS, and fixed with 4% paraformaldehyde in PBS pH 7.4 for 15 min at RT. Cells were washed three times with PBS, permeabilized with 0.1% Triton X-100 in PBS for 15 min, and incubated in a blocking solution containing 10% BSA in PBS for 1 h at RT. F-actin staining was performed overnight at 4°C by incubating samples with rhodamine-phalloidine (1:500, Sigma-Aldrich, P1951) in 1% BSA in PBS. Before mounting using VectaShield (Vector Laboratories), samples were washed five times with PBS.

For live cell imaging, CellTrace Violet-labeled Ben-Men-I cells were grown for 24 h on chambered coverglass (Nunc Lab-Tek, 154461) at a densitiy of 5 × 10^3^ cells/well in 1 mL of culture medium before addition of CFSE-labeled apoptotic bodies. Z-stack confocal images (Zeiss, LSM710Meta equipped with live cell imaging chamber) were acquired every 15 min.

For indirect immunofluorescence, cells were grown on glass cover slips, fixed at RT with 4% paraformaldehyde in PBS for 15 min before permeabilization with 0.1% Triton X-100 in PBS for 15 min and blocking (10% BSA in PBS) for 1 h at RT. Incubation with primary antibodies against LAMP-1 (1:100, abcam, ab25630) or EEA-1 (1:500, abcam, ab70521) was performed overnight at 4°C in 1% BSA in PBS. Samples were washed five times, incubated for 2 h with secondary antibody Alexa Fluor® 546 goat anti-mouse IgG (1:500, Molecular Probes, A11030), and washed three times with PBS, couterstained using DAPI, before mounting with VectaShield.

To estimate phagosomal pH, cells were allowed to internalize particles and were labeled with 50 nM LysoTracker Deep Red (Molecular Probes, L12492) for 15 min before fixation with 4% paraformaldehyde in PBS and analysis by fluorescence microscopy.

### Detection of cytokines/chemokines

Ben-Men-I cells were seeded at a concentration of 6 × 10^4^/mL in DMEM medium and incubated with unlabeled apoptotic cells. After 24 h, the culture supernatants were harvested, centrifuged at 1,000 rpm for 5 min, and assayed for IL-6, IL-8 (Orgenium Laboratories), IL-1RA (R&D, DRA00B), IL-16 (RayBiotech, ELH-IL16-001), MIF (RayBiotech, ELH-MIF-001), or CXCL1 (R&D, DRG00) by ELISA. Cytokines present in apoptotic body preparations were measured and background subtracted. The assays were performed according to the manufacturers’ instructions. Absorbance was read on a Spectramax GEMINI XS microplate reader (450 nm). For multiplexed detection of cytokines/chemokines, the human cytokine array panel A (R&D, ARY005) was used according to manufacturers’ recommendations.

### Statistical analyses

The program R [17] was employed for statistical analyses. One-way ANOVA was used to compare different groups. In case ANOVA indicated significance, significance levels were determined using pair-wise Student’s t-tests with *P* values adjusted according to Holm. Significance is reported as follows: *P* <0.1 marked with #; *P* <0.05 marked with *; *P* <0.01 marked with **; *P* <0.001 marked with ***.

## Results

**Figure 1:**
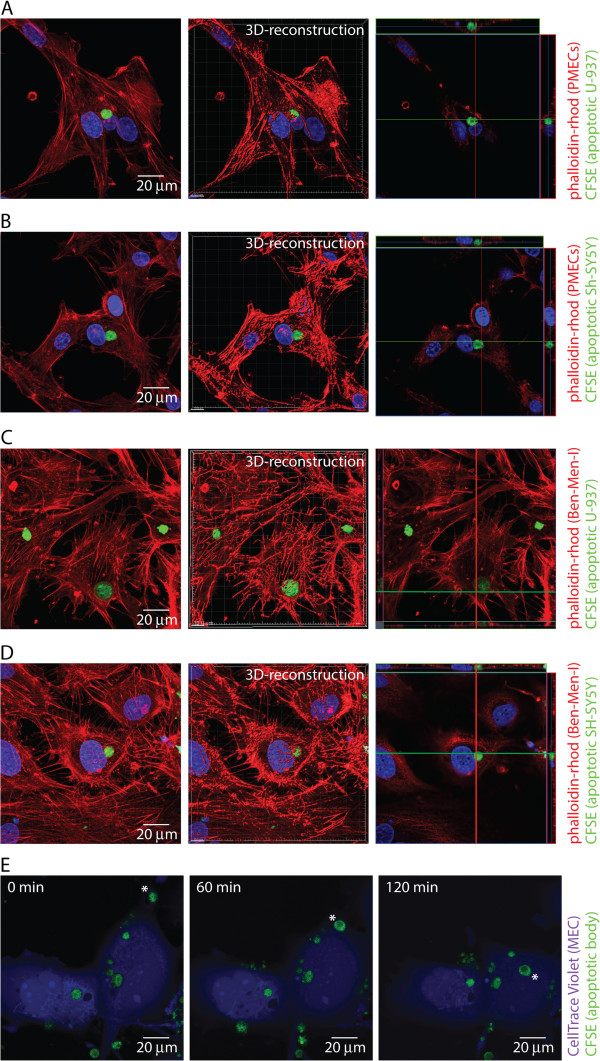
**MECs take up apoptotic cells.** Primary porcine MECs were incubated with CFSE-labeled apoptotic U-937 **(A)** or SH-SY5Y **(B)** cells for 4 h at a MEC:apoptotic body ratio of 1:5. Fixed MECs were permeabilized, actin was stained using phalloidine-rhodomine, and cells were analyzed by confocal microscopy. The right panel represents a 3D-reconstruction and a XYZ-view of the left panel. Ben-Men-I cells were incubated with CFSE-labeled apoptotic U-937 **(C)** or SH-SY5Y **(D)** cells for 4 h at a MEC:apoptotic body ratio of 1:5 and analyzed as above. **(E)** CellTrace Violet-labeled live Ben-Men-I cells were incubated with CFSE-labeled apoptotic U-937 cells and confocal images were taken at 0, 60, and 120 min. The pictures show a representative time course of apoptotic body uptake (marked with *) by MECs.

**Figure 2:**
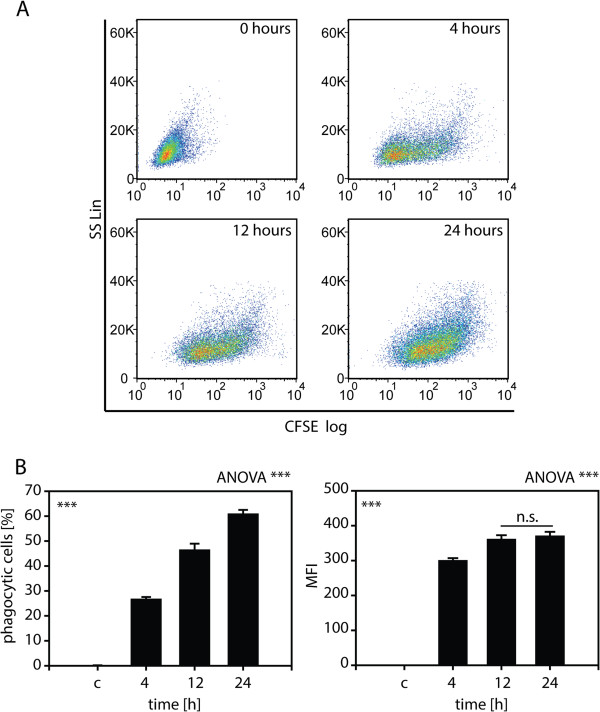
**Time-dependent uptake of apoptotic bodies by MECs. (A)** Ben-Men-I cells were incubated with CFSE-labeled apoptotic U-937 cells for 4, 12, or 24 h at a MEC:apoptotic body ratio of 1:5. CFSE-fluorescence was analyzed using flow cytometry. The bar graphs **(B)** depict the percentage of CFSE-positive MECs (left graph) and the mean fluorescence intensity (MFI - right graph) of three independent experiments with the error bars representing SD. Statistical significance was analyzed by one-way ANOVA. Pair-wise Student’s *t*-test revealed *P* <0.001 adjusted according to Holm for all comparisons (marked ***) except where noted otherwise (n.s. *P* >0.05).

Primary meningothelial cells derived from porcine optic nerves (PMECs) [16] were incubated with apoptotic bodies derived from monocytic U-937 or neuron-like SH-SY5Y cells and the uptake of apoptotic bodies was analyzed by confocal microscopy (Figure 1) as well as flow cytometry (Additional file 1: Figure S1). Primary MECs were found to actively ingest apoptotic bodies derived from monocytic (Figure 1A) as well as neuronal-like cells (Figure 1B). For further studies, Ben-Men-I cells were employed as model for meningothelial cells as they closely resemble MECs (Additional file 1: Figure S2, [6]). To analyze the capacity of MECs to ingest apoptotic cells, Ben-Men-I cells were incubated with CFSE-labeled apoptotic monocyte-like U-937 (Additional file 1: Figure S3A, B) or neuron-like SH-SY5Y cells (Additional file 1: Figure S3C), and the uptake of these apoptotic bodies (Additional file 1: Figure S3D) by MECs was analyzed using confocal microcopy and flow cytometry. As shown in Figure 1, incubation of Ben-Men-I cells with apoptotic bodies derived at a ratio of 1:5 resulted in the engulfment of apoptotic cells by MECs (Figure 1C,D). The uptake of apoptotic cells by MECs seems to be a two-step process with a binding phase where apoptotic bodies are situated on the outside of the plasma membrane of MECs and an engulfment phase as shown using time lapse live-cell confocal microscopy (Figure 1E). To further define the time course of apoptotic cell uptake by MECs, Ben-Men-I cells were incubated for 4, 12, and 24 h with a five-fold excess of apoptotic U-937 cells. Flow cytometric analysis revealed a strong time-dependence of apoptotic cell uptake from 26.5 +/- 1.1% to 60.7 +/- 1.8% apoptotic cell-positive MECs at 4, 12, and 24 h, respectively (Figure 2A and B). Along with the increased number of phagocytotically active cells at later time points (Figure 2B, left graph), the amount of apoptotic bodies taken up also increases over time as evident by increased mean fluorescence intensity (MFI) at the 12-h time point and reaches a plateau after 24 h (Figure 2B, right graph).

**Figure 3:**
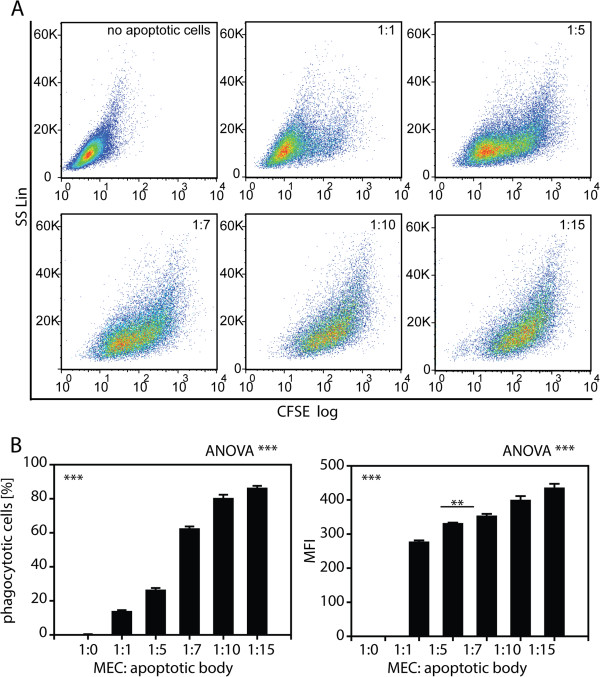
**Dose-dependent uptake of apoptotic bodies by MECs. (A)** Ben-Men-I cells were incubated with CFSE-labeled apoptotic U-937 cells for 4 h at a MEC:apoptotic body ratio of 1:0, 1:1, 1:5, 1:7, 1:10, and 1:15. CFSE-fluorescence was analyzed using flow cytometry. The bar graphs **(B)** depict the percentage of CFSE-positive MECs (left graph) and the mean fluorescence intensity (MFI - right graph) of three independent experiments with the error bars representing SD. Statistical significance was analyzed by one-way ANOVA. Pair-wise Student’s *t*-test revealed *P* <0.001 adjusted according to Holm for all comparisons (marked ***) except where noted otherwise (** *P* <0.01).

**Figure 4:**
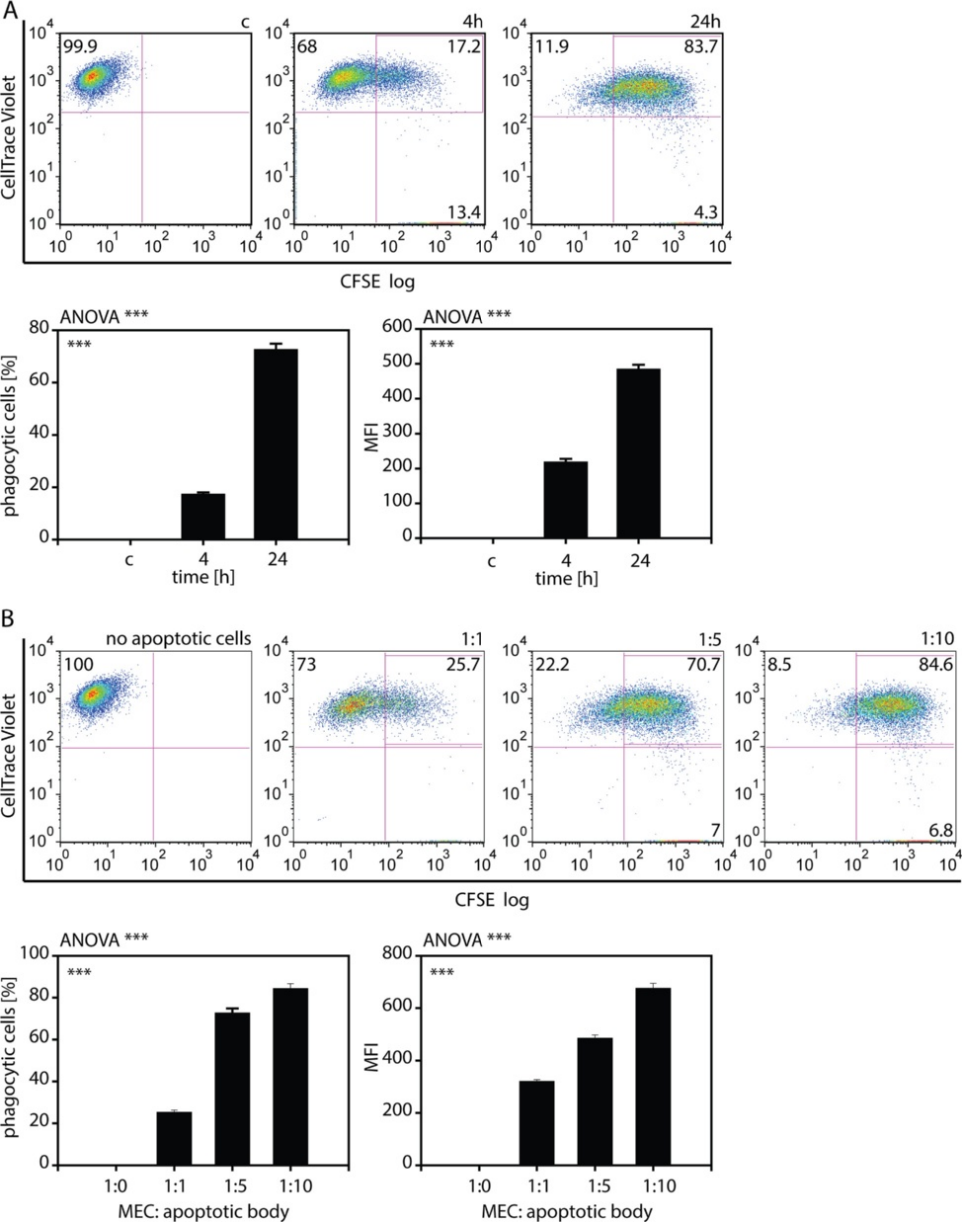
**Uptake of apoptotic neuronal cells by MECs. (A)** CellTrace Violet-labeled Ben-Men-I cells were incubated with CFSE-labeled apoptotic SH-SY5Y cells for 4 (middle panel) and 24 (right panel) h at a MEC:apoptotic body ratio of 1:5. CellTrace violet and CFSE-fluorescence was analyzed using flow cytometry. MECs not incubated with apoptotic bodies (left panel) served as control. **(B)** CFSE-labeled neuroblastoma-derived apoptotic bodies were incubated with CellTrace Violet-labeled Ben-Men-I cells at a ratio of 1:0, 1:1, 1:5, 1:10 for 24 h and cellular uptake was analyzed by flow cytometry. Shown is the average of three independent experiments with error bars depicting SD. Statistical significance was analyzed by one-way ANOVA. Pair-wise Student’s *t*-test revealed *P* <0.001 adjusted according to Holm for all comparisons (marked ***).

Next, to quantify the capacity of MECs for apoptotic U-937 cell uptake, Ben-Men-I cells were incubated with apoptotic cells for 24 h at increasing MEC:apoptotic cell ratios of 1:1, 1:5, 1:7, 1:10, and 1:15, and the amount of engulfed fluorescent U-937 cells was analyzed by flow cytometry. As shown in Figure 3, MECs displayed a dose-dependent increase of apoptotic U-937 uptake with around 15% of positive cells 1:1 ratio and about 85% of phagocytotically active MECs at the 1:10 and the 1:15 MEC:apoptotic cell ratio. To analyze the uptake of apoptotic neuronal cells by MECs, Ben-Men-I cells were incubated with dying SH-SY5Y cells for 4 and 24 h as well as different MEC:apoptotic cell ratios of 1:1; 1:5, and 1:10, respectively. As for U-937-derived apoptotic bodies, Ben-Men-I cells displayed a time- (Figure 4A) and dose-dependent (Figure 4B) uptake of SH-SY5Y-derived apoptotic bodies. While 17.5 +/- 0.6% of MECs were phagocytotically active at the 4-h time point, 72.8 +/- 2.1% of Ben-Men-I cells were capable of ingesting dying neuronal cells at 24 h. Also as with apoptotic U-937 cells, MECs displayed a high capacity for the uptake of apoptotic neuronal cells as indicated by the increased MFI at 24 h compared to 4 h and the high number of phagocytotically active cells even at intermediate MEC:apoptotic body ratios.

**Figure 5:**
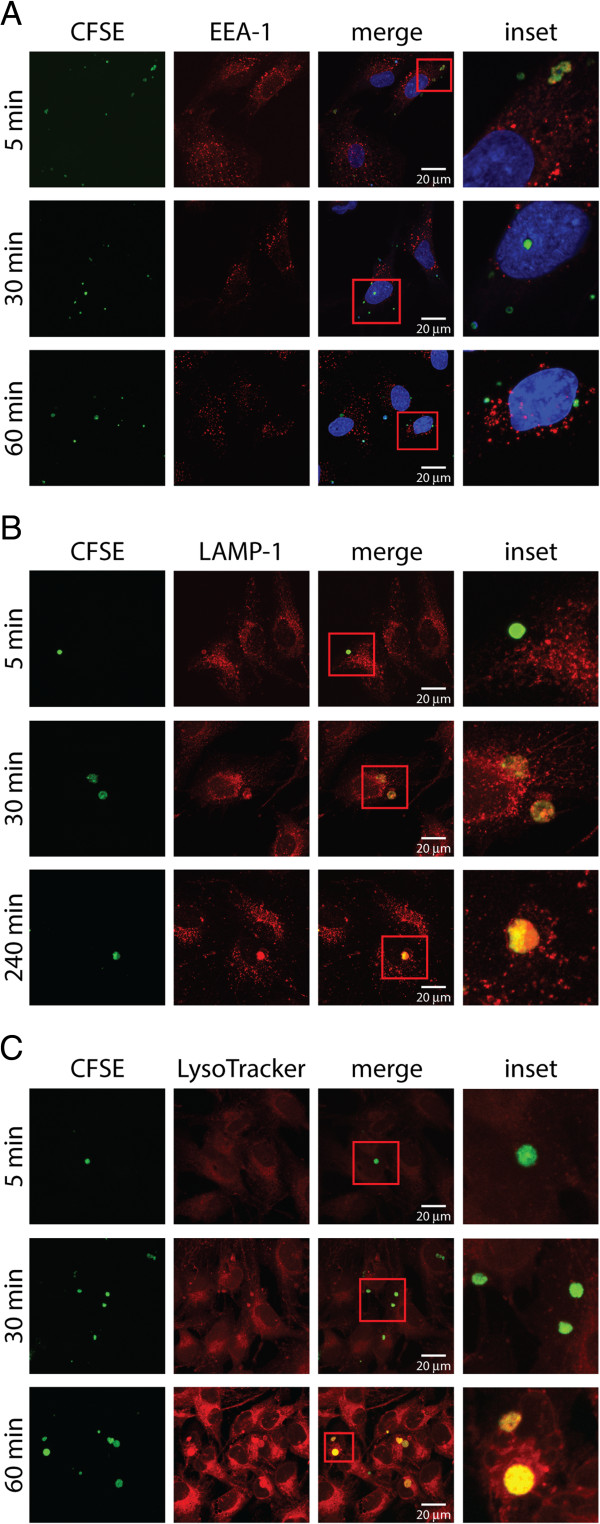
**Apoptotic bodies are taken up by MECs into the lysosomal compartment.** Ben-Men-I cells were pulsed with CFSE-labeled apoptotic U-937 cells for 5 min before fixation at the indicated time point. Subcellular localization of apoptotic bodies inside MECs was analyzed using confocal microscopy at the indicated time points by staining against the early endosomal marker EEA-1 **(A)**, the late endosomal/lysosomal marker LAMP-1 **(B)**, or by using LysoTracker **(C)** as marker for acidified lysosomal compartments. Please note the co-localization of apoptotic bodies (labeled green) with the early endosomal marker EEA-1 (red) at early time points as well as the overlap between apoptotic cells and lysosomal markers (LAMP-1 and LysoTracker - red) in later time points.

To analyze the fate of apoptotic bodies following ingestion by MECs, co-localization studies between markers of the early and late endocytic as well the lysosomal compartment were performed. To this end, MECs allowed to ingest apoptotic bodies for 5 min were fixed at the indicated time points and were stained for the early endocytic marker early endosome antigen 1 (EEA-1), the late endosomal/lysosomal marker lysosomal-associated membrane protein 1 (LAMP1), or the lysotropic and pH-indicating dye LysoTracker. Apoptotic bodies could already be found 5 min after ingestion in EEA-1 positive vesicles (Figure 5A), while LAMP1 was evident 30 min following ingesting co-localization between apoptotic bodies (Figure 5B). As evident by LysoTracker staining and consistent with LAMP1 co-localization, acidification of apoptotic body-containing vesicles was observed at the 30-min as well as 60-min time points (Figure 5C). These data are consistent with the uptake of apoptotic bodies by MECs and their subsequent digestion in the lysosomal compartment.

**Figure 6:**
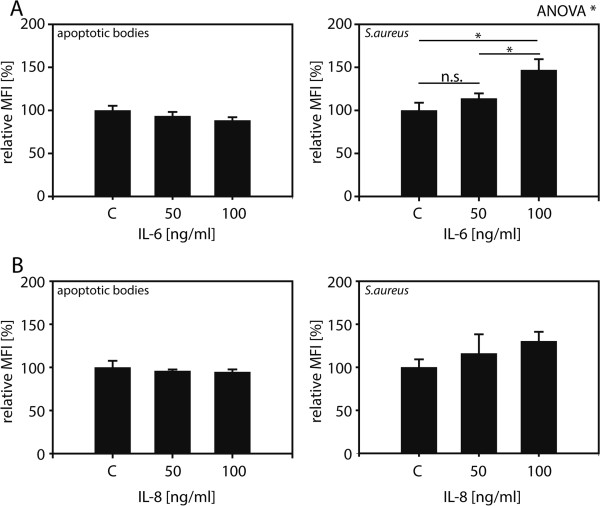
**Bacterial but not apoptotic body uptake by MECs is increased following pro-inflammatory treatment.** Ben-Men-I cells treated with 0, 50, or 100 ng/mL of IL-6 **(A)** or IL-8 **(B)** were incubated with CFSE-labeled apoptotic U-937 (left graphs) or fluorescently-labeled *S. aureus* (right graphs) and particle uptake was determined using flow cytometry. Shown is the average of three independent experiments with the error bars representing SD. Statistical significance was analyzed by one-way ANOVA. *P* values were determined using pair-wise Student’s *t*-test adjusted according to Holm (* *P* <0.05).

MECs are active participants in immunological processes in the brain via cytokine and chemokine secretion. To assess whether ingestion of apoptotic bodies modulates this role, the response to cytokine treatment of MECs as well as cytokine and chemokine secretion by MECs was measured. Treatment with increasing concentrations of IL-6 did not significantly influence the ability of MECs to ingest apoptotic bodies (Figure 6A), while the same treatment was able to increase ingestion of Gram-positive *S. aureus* bacteria, a known substrate for MEC phagocytosis [10]. As for treatment with IL-8, no alteration in the ability of MECs to ingest apoptotic bodies was observed between untreated and IL-8 treated cells, while IL-8 slightly increased the ingestion of *S. aureus* particles (Figure 6B).

**Figure 7:**
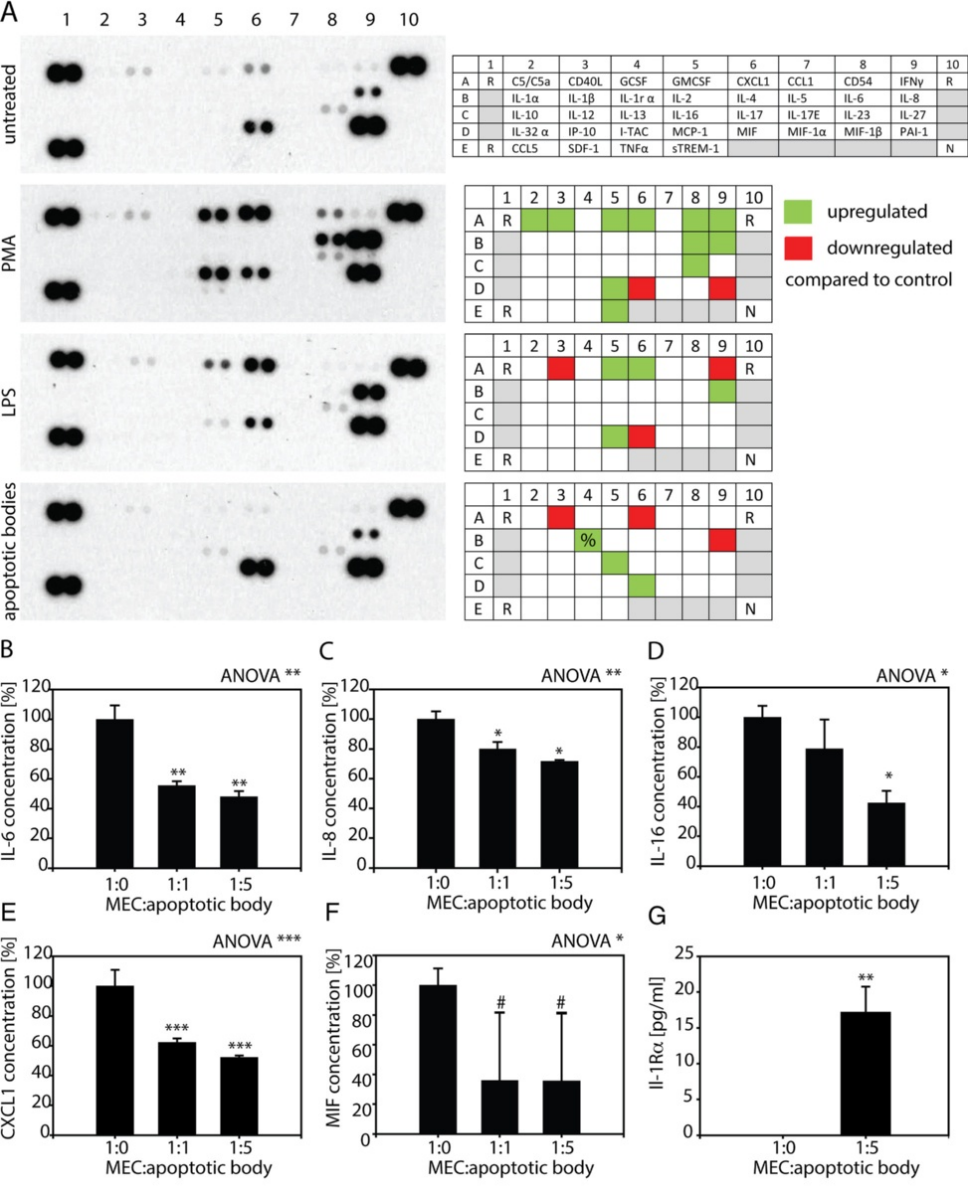
**MECs alter their cytokine and chemokine secretion profile upon uptake of apoptotic bodies. (A)** Ben-Men-I cells were treated with PMA, LPS, and left untreated or were incubated with unlabeled apoptotic U-937 cells at a ratio of 1:5 for 24 h. Cell culture supernatants were analyzed for 36 different cytokines and chemokines using an antibody array. The array is organized in five rows (A-E) and 10 columns (1-10) with two spots for each analyte (R = positive control, N = negative control). Please note that spots corresponding to IL-1 receptor antagonist (marked with %) were weak but distinguishable to the eye. Differences in cytokine secretion between control and treatment groups are depicted in qualitative heat maps. **(B)** Ben-Men-I cells were incubated with unlabeled apoptotic U-937 cells at a ratio of 1:1 or 1:5 for 24 h and the concentration of IL-6 in culture supernatant was measured by ELISA. IL-6 was measured in supernatant of apoptotic cells and subtracted as background from MECs treated with apoptotic cells. Shown is the average of three independent experiments with the error bars representing SD. Statistical significance was assessed using Student’s *t*-test (*P <0.05). Ben-Men-I cells were treated as in **(B)** and with concentrations of **(C)** IL-8, **(D)** Il-16, **(E)** CXCL1, **(F)** MIF (# represents *P* = 0.067), and **(G)** IL-1 receptor antagonist were measured by ELISA. Shown is the average of three independent experiments with the error bars representing SD. Statistical significance was analyzed by one-way ANOVA. *P* values were determined using pair-wise Student’s *t*-test adjusted according to Holm (# *P* <0.1; * *P* <0.05; ** *P* <0.01; *** *P* <0.001). Marked are the comparisons between the control (1:0) and treatment groups.

Uptake of apoptotic cells by professional phagocytes results in alterations of their cytokine and chemokine profile. To assess whether MECs resemble professional phagocytes in this aspect, their cytokine profile in response to apoptotic cell uptake, phorbol 12-myristate 13-acetate (PMA), or lipopolysaccharide (LPS) treatment was analyzed. To this end, supernatants of MECs treated with PMA or LPS or allowed to ingest apoptotic cells were analyzed for 36 different cytokines and chemokines using a multiplexed, antibody array (Figure 7A). Compared to untreated control cells, treatment with PMA known to impact cytokine production caused the upregulation of C5/C5a, CD40L, GMCSF, CXCL1, CD54, IFN-γ, IL-6, IL-8, IL-17E, MCP-1, and sTREM-1, while MIF and PAI-1 secretion was diminished. Treatment of MECs with LPS known to elicit a pro-inflammatory response caused the increased secretion of GMCSF, CXCL1, IL-8, and MCP-1, while CD40L, IFN-γ, and MIF production was decreased. As for cytokine production following apoptotic body uptake, secretion of IL-1 receptor antagonist, IL-16, and MIF was increased, while CD40L, CXCL1, and IL-8 production by MECs was diminished.

To confirm observations made using the multiplex cytokine antibody array and to assess changes of candidate cytokines, levels of cytokines were independently determined using specific ELISA (Table 1). Although below detection level in the cytokine array in control cells, but based on our previous observations, levels of IL-6 in control cells and following uptake of apoptotic bodies was analyzed and found to significantly decrease to 55.6 +/- 4.8% and 48 +/- 3.7% following ingestion of apoptotic cells at ratios of 1:1 or 1:5, respectively (Figure 7B, Additional file 1: Table S1). To control for cytokine production by non-apoptotic U-937 cells in our preparations of apoptotic bodies, IL-6 produced in these apoptotic body preparations was measured at 10 +/- 6% of control and subtracted as background. Similarly, ingestion of apoptotic cells did blunt IL-8 (Figure 7C, Additional file 1: Table S1), IL-16 (Figure 7D, Additional file 1: Table S1), CXCL1 (Figure 7E, Additional file 1: Table S1), and MIF (Figure 7F, Additional file 1: Table S1) production by MECs. Again, measurement of cytokine content in the supernatant of apoptotic cells was performed and was subtracted as background. Incubation of MECs at ratios of 1:1 and 1:5 decreased IL-8 secretion to 80 +/- 8.1% and 71.8 +/- 4.3%, IL-16 secrection to 78.9 +/- 19.4% and 42.46 +/- 8.3%, CXCL1 secretion to 62.2 +/- 2.7% and 52.2 +/- 1.3%, while secretion of MIF dropped to 36 +/- 45.8% and 35.7 +/- 45.6%, respectively. As for the anti-inflammatory IL-1 receptor antagonist, incubation of MECs with apoptotic bodies at a 1:5 ratio resulted in a strong increase from below detection limit in control cells to 15.9 +/- 3.5 pg/mL in MECs ingesting apoptotic bodies (Figure 7G). As for monocyte-derived apoptotic cells, the uptake of neuroblastoma-derived apoptotic bodies also caused altered cytokine secretion of MECs and a decrease in IL-6 and IL-8 secretion by MECs was observed (Additional file 1: Figure S5).

**Table 1:** Uptake of apoptotic bodies alters cytokine secretion profile of MECs

**Cytokine (pg/mL)**	**MEC:apoptotic body ratio**		
	**1:0**	**1:1**	**1:5**
IL-6	13.8 +/- 2.2	9.1 +/- 0.5	8.5 +/- 0.7
IL-8	368.2 +/-32.8	294.6 +/-29.9	264.2 +/-15.6
IL-16	117.8 +/- 15.6	92.9 +/- 31.3	50 +/-7
CXCL1	62.9 +/- 6.8	39.1 +/- 1.7	32.8 +/- 0.8
MIF	89 +/- 17.3	32 +/-25.4	31.7 +/-25.1

## Discussion

Phagocytosis is a complex cellular event by which large particles are actively recognized, engulfed, and degraded [18]. Phagocytosis is performed by professional phagocytes such as macrophages, neutrophils, or dendritic cells, but other cells such as fibroblasts, epithelial, endothelial, and smooth muscle cells are also capable of taking up particulate matter [19-21]. The main function of phagocytosis is the clearance of unwanted material, for example, pathogens from the body as part of host defense mechanisms. However, besides the removal of pathogens, the orderly disposal of apoptotic cells as they occur during tissue remodeling, normal cell turnover or in the aftermath of a successfully battled infection is achieved via phagocytosis [21]. Therefore it is conceivable that MECs and their ability to clear apoptotic cells might be important during tissue remodeling processes in the brain for example during neurodegenerative disorders. While microglia is known to clear apoptotic neuronal cells [22], under certain circumstances also MECs might assist in this task. As MECs form a tight monolayer and are to our knowledge not motile, MECs might only clear dying cells located on the periphery of the neuronal tissue in close proximity to MECs covering the pia mater. Therefore, such a mechanism might be more prevalent in areas of the CNS such as the optic nerve with its small volume compared to MEC surface than in other parts of the brain with a larger volume of neuronal tissue. Interestingly, MECs were shown to react to pathological conditions during the course of glaucomatous optic nerve degeneration [8]. Aside from apoptotic cells inside neuronal tissue, all apoptotic bodies somehow entering the CSF compartment are likely subject to phagocytosis by MECs, as the specialized architecture of the subarachnoidal space with its extensive trabeculae and septa covered by MECs provides a large surface, thus greatly boosting the contact interface between these cells and the CSF. Although mounting evidence supports phagocytosis by MECs [5,6,10], whether these cells perform uptake of apoptotic bodies *in situ* is unclear and further *in vivo* studies beyond the scope of this study are necessary to address this question.

Beyond this potential role of MECs in tissue remodeling, removal of apoptotic cells by MECs is likely important to prevent the accumulation of apoptotic debris which might lead to autoimmunity due to the increased presence of autoantigens as observed, for example, during systemic lupus erythematosus [23]. We previously showed a pro-inflammatory reaction of MECs where stimulation with the Gram-negative bacteria-derived danger signal lipopolysaccharide resulted in the increased secretion of IL-6 and IL-8 [5]. Furthermore, we implicated MECs in the clearance of Gram-positive and Gram-negative bacteria from the CSF compartment, together establishing an immunological role for MECs in the CNS [10]. Now we find that MECs are also capable of ingesting apoptotic cells via the endocytotic pathway and that uptake leads to a decrease of IL-6, IL-8, IL-16, MIF, and CXCL1 production, while levels of IL-1 receptor antagonist, are increasing. IL-6 [24], IL-8 [25], CXCL1 [26], MIF [27], and IL-16 [28] are known to act pro-inflammatory or immune response stimulating, while IL-1 receptor antagonist [29] is known as anti-inflammatory cytokine. As MECs are capable of secreting various pro-inflammatory cytokines in response to PMA treatment or following exposure to LPS via an NF-kB-dependent mechanism as reported by us [5] supporting a role for these cells during inflammation, the alteration of cytokine and chemokine production following apoptotic body uptake suggests that MECs can acts also in an anti-inflammatory manner. Thus, the downregulation of pro-inflammatory IL-6, IL-8, IL-16, MIF, and CXCL1 together with the upregulation of anti-inflammatory IL-1 receptor antagonist following uptake of apoptotic cells is consistent with an anti-inflammatory role of MECs.

Thus, the data presented here together with previous findings of a pro-inflammatory role of MECs point to an integral role of MECs in immunological processes in the CNS, both in the initiation and the shutdown of immune reactions. Following infiltration of the CSF compartment by immune cells during the course of an infection, clearance of such cells and the shut-down of the immune reaction is likely especially important in the CNS, as unchecked immunological processes might lead to serious neurological damage.

Taken together, besides their clear role in physically enveloping and protecting the CNS and their function in the immunological protection of the brain during bacterial infection, MECs might be involved in clearing apoptotic cells during neurodegenerative disorders and are likely important for keeping immunological defense mechanisms in the CSF through the secretion of anti-inflammatory cytokines.

## Abbreviations

ANOVA: Analysis of variance; CXCL1: Chemokine cc-x-c motif ligand 1; CSF: Cerebrospinal fluid; CNS: Central nervous system; EEA-1: Early endosome antigen 1; ELISA: Enzyme-linked immunosorbent assay; GMCSF: Granulocyte macrophage colony-stimulating factor; IFN-γ: Interferon gamma; IL: Interleukin; LAMP1: Lysosomal-associated membrane protein 1; LPS: Lipopolysaccharide; MECs: Meningothelial cells; MFI: Mean fluorescence intensity; MIF: Macrophage inhibitory factor; MCP-1: Monocyte chemoattractant protein-1; PAI-1: Plasminogen activator inhibitor 1; PMA: Phorbol 12-myristate 13-acetate; PMECs: Primary porcine meningothelial cells; sTREM-1: Soluble triggering receptor expressed on myeloid cells 1.

## Competing interests

The authors declare that they have no competing interests.

## Authors’ contributions

JL performed experiments, analyzed data, and revised the manuscript. LF performed experiments and revised the manuscript. PM conceived the study and revised the manuscript. HEK conceived the study and revised the manuscript. JF conceived the study and revised the manuscript. AN conceived the study, analyzed data, and wrote the manuscript. All authors read and approved the final manuscript.

## Supplementary Material

Additional file 1: Figure S1 Uptake of apoptotic monocytic and neuronal apoptotic bodies by primary MECs. **Figure S2.** Ben-Men-I cells are a faithful model for MECs. **Figure S3. (A)** Labeling of cells using CFSE. U-937 cells were labeled using CFSE and labeling efficiency was determined using FACS analysis to be around 99%. **Figure S4.** Size-difference is sufficient to distinguish between apoptotic U-937 and Ben-Men-I cells. **Figure S5.** PMECs were incubated with unlabeled apoptotic SH-SY5Y cells at a ratio of 1:1 or 1:5 for 24 hours and the concentration of IL-6 **(A)** and IL-8 **(B)** in culture supernatant was measured by ELISA. Click here for file
