# Supplementary material for: Anti-inflammatory response following uptake of apoptotic bodies by meningothelial cells
Source: J Neuroinflammation. 2014 Feb 24;11:35. doi: 10.1186/1742-2094-11-35 (PMC3941933; doi:10.1186/1742-2094-11-35)
Supplement: Additional file 1: Figure S1 — Uptake of apoptotic monocytic and neuronal apoptotic bodies by primary MECs. Figure S2. Ben-Men-I cells are a faithful model for MECs. Figure S3. (A) Labeling of cells using CFSE. U-937 cells were labeled using CFSE and labeling efficiency was determined using FACS analysis to be around 99%. Figure S4. Size-difference is sufficient to distinguish between apoptotic U-937 and Ben-Men-I cells. Figure S5. PMECs were incubated with unlabeled apoptotic SH-SY5Y cells at a ratio of 1:1 or 1:5 for 24 hours and the concentration of IL-6 (A) and IL-8 (B) in culture supernatant was measured by ELISA. [file 1742-2094-11-35-S1.pdf]

Supplemental Figures

Figure S1: Uptake of apoptotic monocytic and neuronal apoptotic bodies by primary MECs. Primary porcine MECs were incubated with CFSE-labeled apoptotic U-937 or SH-SY5Y cells at a ratio of 1:1 for 24 hours. Uptake of labeled apoptotic cells was performed by FACS analysis. Shown in one representative experiment out of three (A). The gate represents CFSE-positive PMECs. The bar graphs (B) depict the percentage of CFSE-positive PMECs (left graph) and the mean fluorescence intensity of CFSE-positive PMECs (right graph).

Figure S2: Ben-Men-I cells are a faithful model for MECs. **(A)** Ben-Men-I cells express desmoplakin. Ben-Men-I cells were fixed and stained using mouse anti-desmoplakin antibodies (1:50, Abcam ab16434). **(B)** Ben-Men-I cells express the epithelial cell marker epithelial membrane antigen (EMA). Ben-Men-I cells were fixed and stained using mouse anti-EMA antibodies (1:100; Abcam ab28081). Please note that Ben-Men-I cells express the meningothelial cell markers desmoplakin and EMA further confirming that Ben-Men-I cells are a valid model for MECs.

Figure S3: **(A)** Labeling of cells using CFSE. U-937 cells were labeled using CFSE and labeling efficiency was determined using FACS analysis to be around 99%. Shown is a representative experiment (n=3). **(B)** Induction of apoptosis in U-937 cells. 10^6 U-937 cells were treated with 700 ng/ml actinomycin D for 20 hours to induce to apoptosis. Induction of apoptosis was established using Annexin V and propidium iodide (PI) staining. >89% of the cells were Annexin V positive/PI negative indicative for apoptosis, < 10% were Annexin V positive/PI positive indicative for necrotic cells. Shown is one representative experiment of three. **(C)** Induction of apoptosis in SH-SY5Y cells. 10^6 SH-SY5Y cells were treated with 900 ng/ml actinomycin D for 24 hours to induce apoptosis. Cells were analyzed as in B. Following actinomycin D treatment, 55% of SH-SY5Y cells were Annexin V positive/PI negative indicative for apoptosis, while 28.5% of cells were Annexin V positive/PI positive. Shown is one representative experiment of three. **(D)** Apoptotic cells but not live cells are taken up by MECs. MECs were incubated with labeled live and apoptotic U-937 cells and uptake of fluorescently-labeled cells by MECs was analyzed using flow cytometry. Shown is one representative experiment out of three.

Figure S4: Size-difference is sufficient to distinguish between apoptotic U-937 and Ben-Men-I cells. CellTrace Violet-labeled Ben-Men-I cells were mixed at a ratio of 1:5 with CFSE-labeled apoptotic U-937 cells. The cell mixture was analyzed by flow cytometric analysis and events were gated as indicated in the left panel. Based on CellTrace Violet staining (in the middle and right panel), the cell population represented by the gate contains only Ben-Men-I cells or Ben-Men-I cells containing U-937 cells but not free U-937 cells. Using this size-based gating strategy, Ben-Men-I cells can be reliably distinguished from apoptotic U-937 cells. This gating strategy was employed throughout the study whenever the uptake of apoptotic U-937 cells by Ben-Men-I cells was analyzed.

Figure S5: PMECs were incubated with unlabeled apoptotic SH-SY5Y cells at a ratio of 1:1 or 1:5 for 24 hours and the concentration of IL-6 (A) and IL-8 (B) in culture supernatant was measured by ELISA. IL-6 and IL-8 was measured in supernatant of apoptotic cells and subtracted as background from MECs treated with apoptotic cells. Shown is the average of three independent experiments with the error bars representing SD. Statistical significance was assessed using one-way ANOVA and pair-wise Student's t-test ($p < 0.05$ is marked with *; $p < 0.001$ is marked with ***). The table shows concentrations of IL-6 and IL-8 as measured by ELISA.

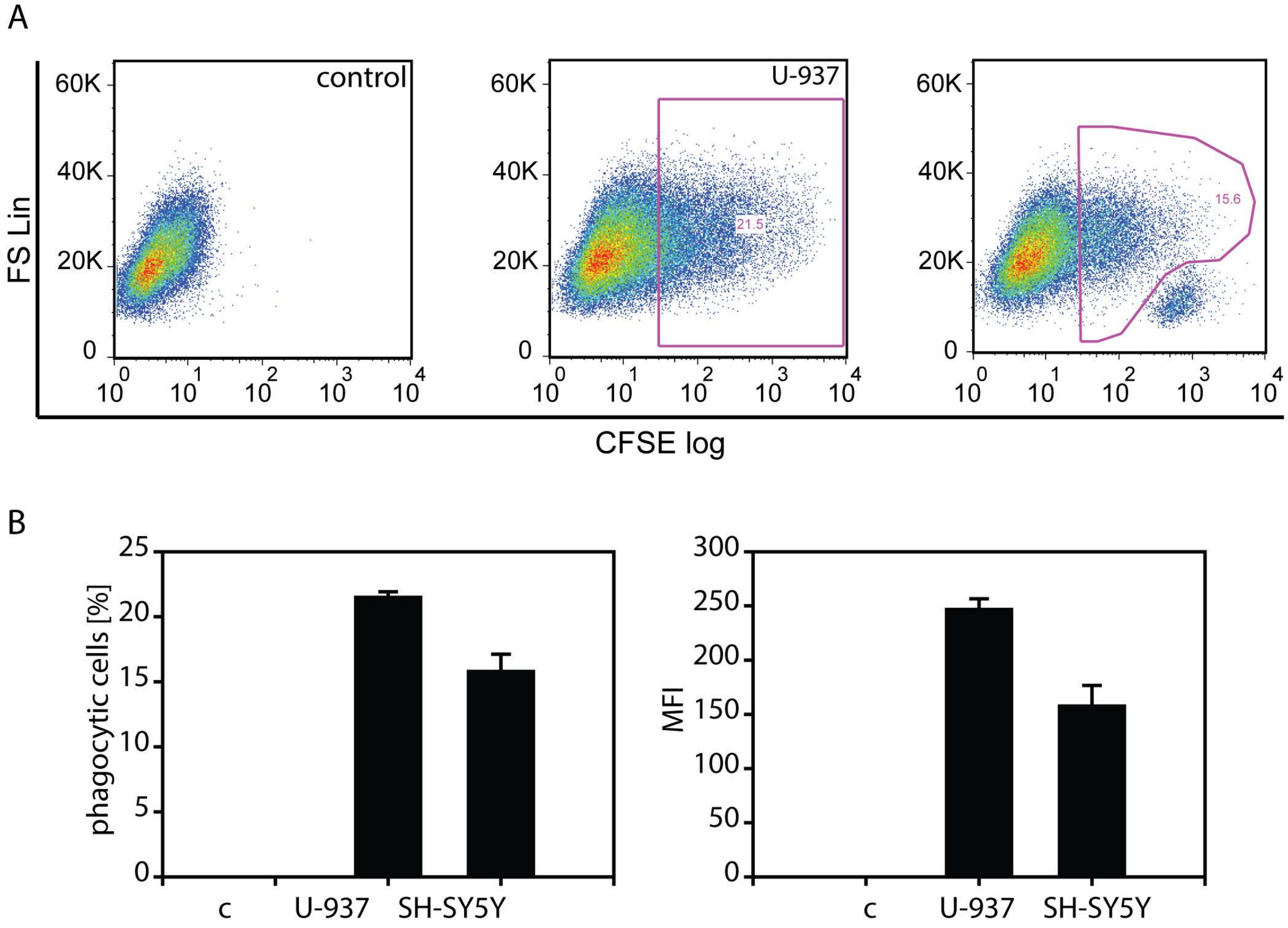

Figure S1 - Li et al.

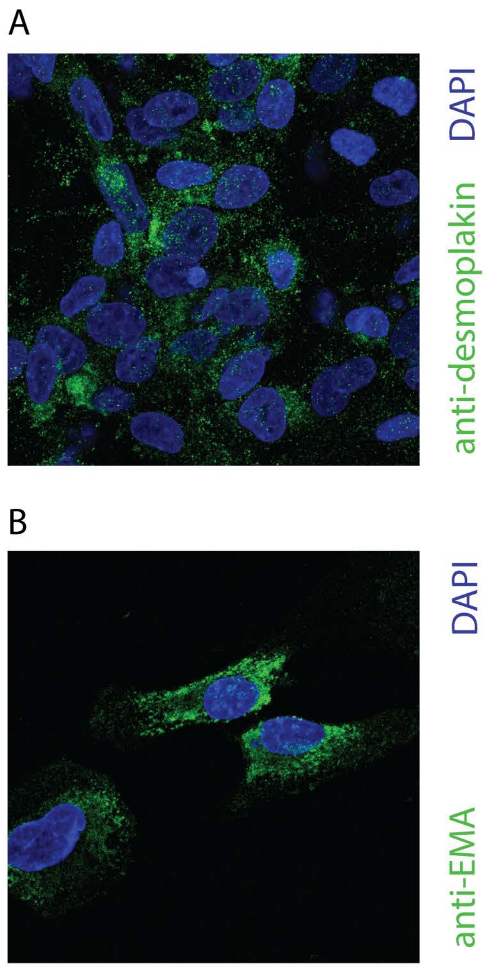

Figure S2 - Li et al.

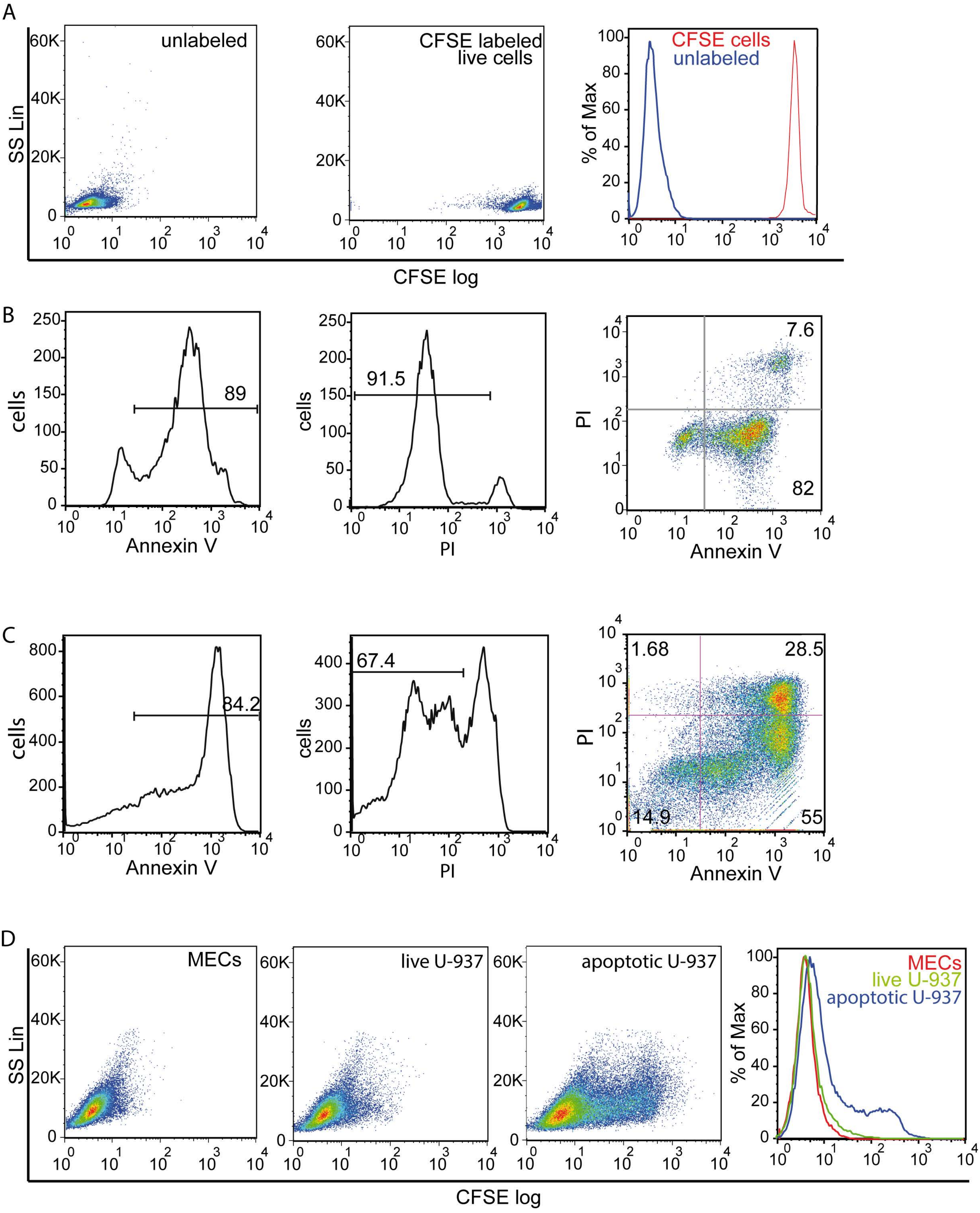

Figure S3 - Li et al.

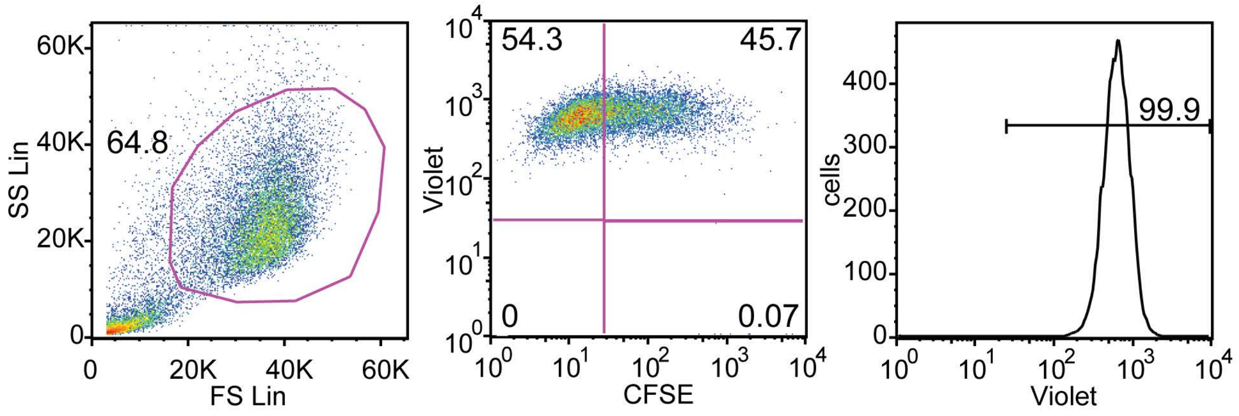

Figure S4 - Li et al.

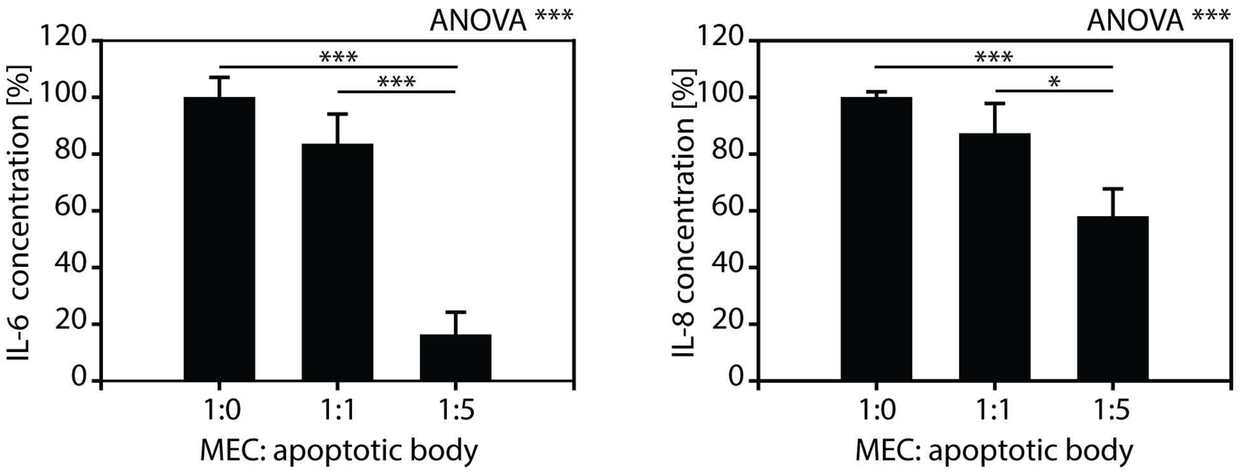

**cytokine (pg/ml)**	**MEC:apoptotic body ratio**		
	1:0	1:1	1:5
**IL-6**	8.8 +/- 0.6	7.4 +/- 0.9	1.4 +/- 0.7
**IL-8**	252.2 +/- 4.9	219 +/- 26.7	146.2 +/- 24.6

Figure S5 - Li et al.
